# Analysis of online user discussions on Reddit associated with the transition of use between HIV PrEP therapy

**DOI:** 10.3389/fpubh.2023.1073813

**Published:** 2023-06-28

**Authors:** Hector Godinez, Qing Xu, Tiana J. McMann, Jiawei Li, Timothy Ken Mackey

**Affiliations:** ^1^S-3 Research, San Diego, CA, United States; ^2^Global Health Policy and Data Institute, San Diego, CA, United States; ^3^Global Health Program, Department of Anthropology, University of California, San Diego, San Diego, CA, United States

**Keywords:** PrEP, HIV/AIDS, drug switching, pharmacovigilance, infoveillance, infodemiology, minority health, social media

## Abstract

In 2019, the U.S. Food and Drug Administration (FDA) approved emtricitabine and tenofovir alafenamide (Descovy) as another option for HIV pre-exposure prophylaxis (PrEP) prevention for high-risk adults and adolescents. With the introduction of this new PrEP, millions of current users on emtricitabine and tenofovir disoproxil fumarate (Truvada), another PrEP medication currently used to prevent HIV transmission, have options of whether to continue their current treatment regime or transition to new treatment options. The objective of this study was to conduct a descriptive analysis to characterize user-generated social media conversations on Reddit associated with FDA-approved PrEP prevention treatment options. Key themes identified were associated with perceptions, knowledge, and attitudes associated with the transition of use of different PrEP medications. Data were collected retrospectively and prospectively from the Reddit platform for posts with keywords filtered for HIV, PrEP, and FDA-approved PrEP prevention treatment from October 2020 to December 2020. We chose the Reddit platform based on prior studies that have identified PrEP user conversations and insights on access challenges for specific AIDS communities, such as gays and men who have sex with men (MSM). Reddit posts were then manually annotated using an inductive content coding approach for key themes regarding the transition of use and other emergent themes from user-generated content. Formal coding of text data was conducted with refined codes, and sub-codes created. A total of 3,120 posts were analyzed from Reddit resulting in 315 posts that were coded for PrEP and 105 posts (33.33%) specific to user discussions regarding the transition of PrEP prevention. Overall, users expressed interest in drug switching to Descovy, particularly in the context of poorer adherence or concerns about existing side effects associated with Truvada. Other major themes included discussions about the cost of Descovy, apprehension about side effects in comparison to Truvada, insurance coverage changes, and discussions about the donation of Truvada to other users after transitioning. Among these discussions, topics related to sexual minorities, including MSM, reported concerns when considering a switch in their HIV prevention regime. Understanding the changing public perception associated with the introduction of new HIV prevention is important in the context of market access, patient safety, pharmacovigilance, and health equity, particularly among high-risk populations such as MSM. Results support the use of social media from a digital pharmacovigilance perspective to better understand emerging HIV prevention, treatment, and adherence challenges experienced by patients.

## 1. Introduction

In 2020, ~37.7 million people were living with human immunodeficiency virus (HIV) globally, and as of 2021, over 28.2 million people were accessing anti-retroviral therapy (ART), representing an increase of more than 20 million people accessing treatment since 2010 (1). This is in large part due to a large reduction in anti-retroviral therapy (ART) prices as well as increases in the availability of treatment options approved for HIV (2, 3). In addition to ARTs, pre-exposure prophylaxis (PrEP) is a highly effective medication, that when taken as prescribed, is highly effective for preventing HIV infection, including for those at risk through sex or injection drug use (4). Importantly, certain sexual and gender minorities (SGMs), including men who have sex with men (MSM) and transgender women, have been disproportionately impacted by HIV infection and may have different attitudes toward PrEP therapy that can impact uptake and adherence (5, 6). Understanding the willingness to use PrEP therapy among these at-risk SGM populations, including in the context of drug transition and switching behavior, is critical to reducing HIV incidence.

In 2012, emtricitabine and tenofovir disoproxil fumarate (brand name Truvada) was approved by the U.S. Food and Drug Administration (FDA) for use in HIV-negative individuals to reduce the likelihood of contracting HIV, in addition to its use to treat active infections (7, 8). Since Truvada's approval as the first drug for HIV prevention using pre-exposure prophylaxis (PrEP), other drugs have also received approval for a PrEP indication in an oral formulation. In 2019, the FDA approved emtricitabine and tenofovir alafenamide (brand name Descovy) for PrEP in at-risk adults and adolescents (9). Truvada is a tenofovir disoproxil fumarate (TDF), and Descovy is a tenofovir alafenamide (TAF).

Although the chemical composition of these therapies is different, studies have found both formulations to be non-inferior to each other (10). Descovy's approval came with reports that its safety profile might be more favorable, with early evidence of fewer side effects compared to other PrEP options. This factor was considered important, as clinicians cited concerns about side effects when prescribing PrEP (11). However, evidence to date does not support that one drug has better safety and efficacy over the other, with a recent meta-analysis finding a difference in viral suppression and bone and renal side effects only when using a boosting agent with TAF (12). Apart from these considerations, cost and access issues are an ongoing concern as these drugs lacked generic treatment options at certain periods of time (e.g., the FDA approved a generic version of Truvada in June 2017) (9, 13). Hence, the potential benefits of increased competition and patent expiry have also been discussed and anticipated (13). Additionally, medical mistrust among SGMs and other minority populations regarding the effectiveness of PrEP may also represent a critical barrier to uptake and adherence (14–19).

Despite active conversation about the similarities, differences, and possible benefits vs. risks of these different FDA-approved PrEP therapy regimes among the HIV/AIDS community, few studies have specifically examined perceptions, attitudes, and behaviors associated with the transition of use for PrEP, though several studies have been published assessing barriers and facilitators to PrEP therapy as discussed on social media (20–23). Furthermore, few studies have examined social media to characterize how willingness to use PrEP may be impacted by perceptions and attitudes about drug transitions, particularly among SGMs as a specific population of interest. This study adds to this growing body of literature on social media and PrEP by conducting a descriptive analysis identifying and analyzing Reddit posts specific to users reporting their lived experiences with transitions of use attitudes for PrEP prevention therapy, while also examining whether these conversations included users who discussed issues related to or specifically self-identified as racial, ethnic, or sexual/gender-identification minorities (e.g., LGBTQ).

## 2. Method

### 2.1. Data collection

We used the programming language Python™ to develop a custom programming script to collect publicly available posts from Reddit. Posts were collected from Reddit over a 60-day study period (13 October 2020–11 December 2020) and allowed us to collect both retrospective data from posts that occurred prior to the date of collection and prospective data, posts collected starting on the date of collection. This data collection method and time period were chosen as it was deemed sufficient to collect both past and recent conversations regarding PrEP. Specifically, data were collected by retrieving posts from search results and sub-Reddits filtered for common PrEP and HIV-associated keywords including “HIV,” “PrEP,” “Truvada,” and “Descovy.” These keywords led to additional associated keywords and hashtags identified in Reddit conversations relating to HIV prevention and PrEP that were used for additional data collection. We chose the Reddit platform based on prior studies that have identified PrEP user conversations and insights on access challenges for specific AIDS communities, such as gay and MSM.

### 2.2. Data analysis

**Figure 1:**
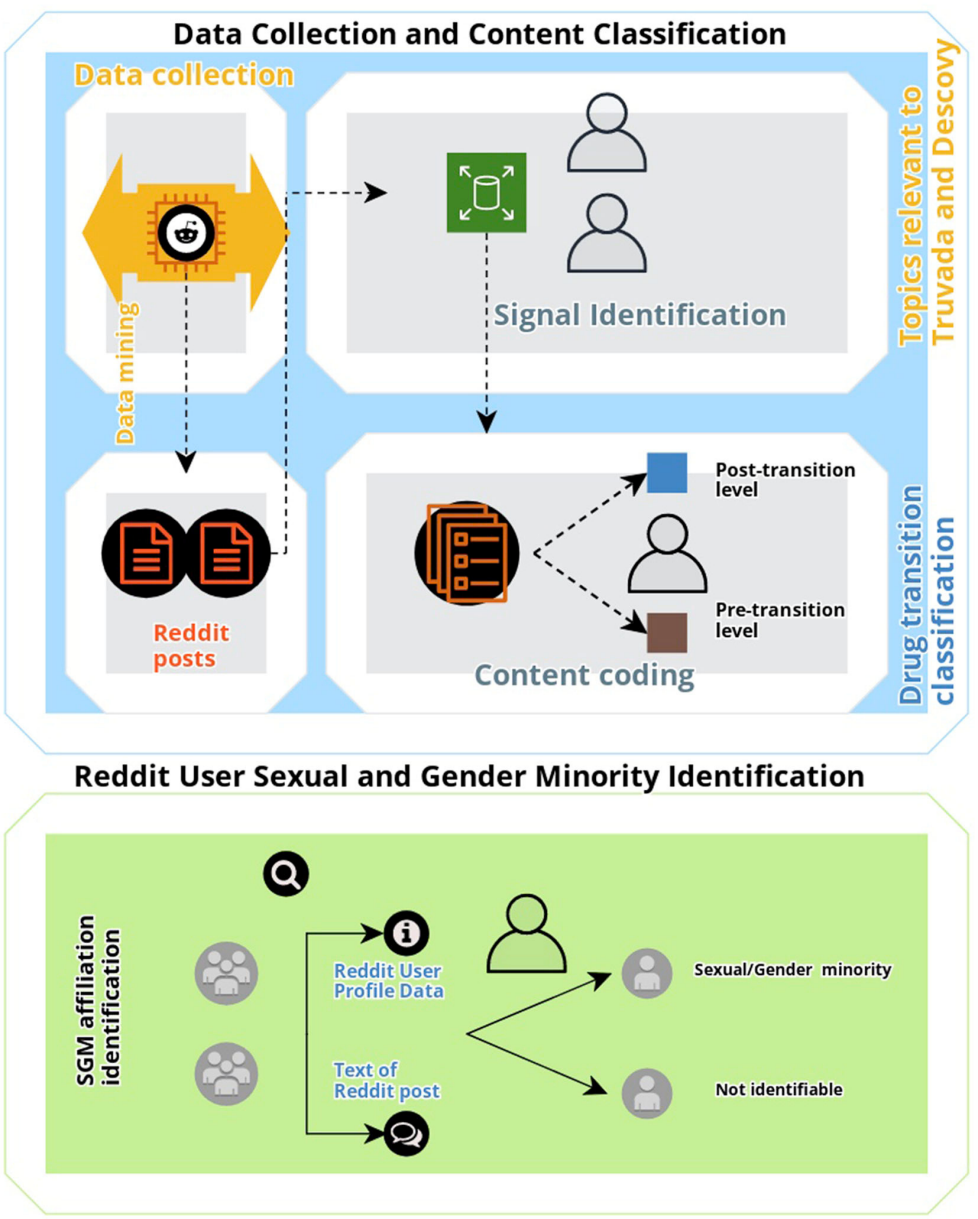
Overview of study methodology.

To identify, characterize, and elucidate conversation associated with the two approved PrEP medications, Truvada and Descovy, we manually annotated all collected posts. First, we identified all signal posts. Posts were deemed as “signal” if they were (a) user-generated (i.e., not posted by organizations or media outlets); and (b) conversation discussing topics relevant to Truvada and Descovy accessibility, use and or transition of use, effectiveness, insurance coverage, and associated barriers. Advertisements of the drug, public announcements, and posts not related to Truvada and Descovy were specifically excluded from analysis as the focus of this study was to identify and characterize user-generated posts regarding behavior associated with PrEP, and with a specific focus on drug transitions, not drug promotion or HIV health education and promotion. To classify the content of Reddit posts, a general inductive approach was utilized to code the textual data for descriptive analysis (24). All identified signal posts were reviewed by the first author (HG) and notes were taken on general themes of posts. Formal coding of text data was conducted with refined codes, and sub-codes were created. A final coded dataset was reviewed by the second author (QX), and differences in code definitions and applications were reconciled by the first and second authors. Inductively derived codes (see Table 1) were then included in the analysis if they included content discussing the two FDA-approved PrEP drugs “Truvada” and “Descovy.” A final coded dataset was reviewed by the first, second, and last authors (TKM) to assess whether any differences in code definitions and application occurred with any differences reconciled by consensus on the correct classification. The first and second authors achieved high inter-coder reliability for signal coding (kappa = 95.32). Additionally, the text and metadata (e.g., gay label and rainbow emoji) of Reddit posts associated with PrEP content that was relevant to the themes generated for this study were also coded to determine whether they included comments or discussions related to SGM issues, or whether users specifically self-identified as gay, lesbian, or bisexual (sexual minority status) or whether they self-reported their expression of their gender identity as different from their sex assigned at birth (gender minority status) (25). A visual summary of the study methodology used is shown in Figure 1.

**Table 1:** Inductive code list and identified sub-codes.

**Topic level**	**Code number**	**Description**	**Examples**	**Total**
Pre-Switch Level (72.38% of all signals)	A-1	Sharing or seeking knowledge regarding potential side effects from Descovy and also discussing reasons why this influenced their decision not to engage in drug transition	“So I got prescribed descovy [sic] today. I haven't taken it yet. Before I do, I just wanted to get everyone's thoughts and opinions on it. Should I expect any real side effects [sic] the first few days? I know it is and I feel stupid for asking but it's completely safe right? Thanks” “I just started PrEP on Truvada like LAST WEEK [sic] and now Descovy is FDA approved. I pinged my doc if I should switch when my current supply of Truvada is gone and she responded by prescribing Descovy. I'm waiting for her to answer the question of [sic] ‘should I switch now?'. I feel like the lower side effects are great but it's also [sic] ‘bleeding edge' for PrEP so I'm not sure how I feel about that. So guys who have been on PrEP longer than I have: Are you switching to Descovy?”	15 (17.65%)
	A-2	Sharing or seeking insurance coverage information associated with drug switching to a new PrEP treatment and why this impacted their decision to transition or not to transition to another PrEP treatment	“I want to get on PrEP before I start having condomless sex with my fwb specifically Descovy since it has less [sic] side effects. However, my insurance company …told me that I will have a co-pay [sic] around $200 for only a 30-day prescription! What should I do?”	11 (12.94%)
	A-3	Asking for or sharing knowledge on the process of switching medication	“What are your experiences with truvada and descovy [sic]? Did you tolerate one better? How did you start the conversation with your doctor? I'd appreciate any advice you can give me.”	24 (22.86%)
	A-4	Interest in switching to Descovy after having side effects from Truvada	“Last night I topped (i almost never have sex) and a guy and the condom broke. I didn't notice until it was over so I don't know how long it was broken for (but the whole thing only lasted like 7 min?)…I went to the doctor to ask about PEP today. She didn't really seem to know what it was (she was a physician's assistant) and went and called the doctor and came back basically saying sure we'll give it to you. I didn't feel really assured by that. I went and got the prescriptions [truvada tivicay (sic)] and took them for the first day so far I feel okay. But I am really afraid of the potential side effects I keep reading about online. Especially the severe shit like liver/kidney failure. Should I be taking this given what my situation is?”	12 (28.23%)
Total				62 (72.94%)
Post Switch Level (21.90% of all signals)	B-1	Sharing experience and reasons for switching from Truvada to Descovy	“I had bad long lasting [sic] side effects with Truvada for PreP so I thought I'd make this post to help others with the same experience with Truvada. With Truvada, I had diarrhea and stomach cramping always 30 min after ingestion. Doctors told me to continue the medication as it would get better with time. Went on for months and it was the same thing. A week ago, I switched over to Descovy, and it's been fantastic compared to Truvada. Only minimal diarrhea for the first 3 days and then nothing. Its [sic] been a week now and I'm not experiencing any side effects. Doctor [sic] informed me it was most likely due to the fact that the tenofovir in Truvada is absorbed by your body in lesser doses than the tenofovir in Descovy is which is absorbed [sic] a higher quantity. Whether this is true or not I have no idea. I just know that I'm experiencing no side effects with Descovy besides the[sic] mild diarrhea the first few days. Hope this helps!”	2 (2.35%)
	B-2	Asking for advice on mix using Truvada and Descovy	“I've been trying to take both but pure for men says to take it 2 h after or before taking any other medication. What is your advice and does pure for men lessen the protection and benefits of descovy [sic]”	6 (7.06%)
	B-3	Asking for advice on managing side effects	“Just started taking descovy [sic] about 2 weeks ago and have been experiencing the worst gas of my life. Has anyone had a similar experience? Also, any advice on how to manage the side effects would be appreciated.” “Seems like I am having the same side effects with my Descovy as I did with Truvada (sucks because I switched because of this). Side effects for me are stomach problems and not being able to put on weight (for me being as thin as I am this is not necessarily a good thing before someone says you should be happy with this side effect).”	11 (12.94%)
	B-4	Asking for advice on donating leftover Truvada	“My gay GP just switched me from Truvada to Descovy not because I've had any side effects but just because my plan would cover it and if Descovy has a lower chance of side effects why not? When he switched me, I told him I'd just refilled my Truvada and he said I'd probably have to wait until the Truvada ran out to go onto the Descovy. OH NO... He sent in the prescription for Descovy and it was ready at my pharmacy the next morning. So now I have a sealed original packaging completely untouched 30-day bottle of Truvada that I will not [sic] ever use. Last I heard these retail for ~$1,800. So, I don't want to just flush it. I live in Los Angeles so I'm sure there's someone in need who could use it! Is there some way I can donate it? Is that even legal? If so, how would I find a place that would take it? If not... any ideas of how I might be able to get it into the hands of someone in need? It is criminally expensive medication so I don't just want to throw it away. All advice welcome PM me if you're more comfortable.”	4 (4.71%)
Total				23 (27.06%)

## 3. Results

### 3.1. Overview

We collected a total of 2,349 unique Reddit posts over the study period. Descriptive analysis and manual annotation of all collected posts identified 85 (4.47% or the entire dataset) signal posts. Posts that did not meet our inclusion criteria for this study but were nevertheless relevant to PrEP facilitators and barriers included posts discussing differences between post-exposure prophylaxis (PEP) and PrEP therapy, confusion regarding PrEP therapy, experiences regarding healthcare providers' lack of PrEP knowledge and training, and comments believing that Descovy advertisements only targeted certain minority populations were excluded. Based on the publicly available text in Reddit posts, the largest representation from a minority group were for members associated with the sexual minority community (95.29%, *n* = 81), specifically gay and MSM. A summary of parent and sub-code book results and corresponding de-identified examples are reported in Table 1.

### 3.2. Data analysis

According to our data analysis, all signal posts were categorized into two main parent-level topics: (i) pre-transition: posts regarding users who currently take Truvada, users recognizing a new PrEP medication has been approved, and users sharing or seeking knowledge regarding the differences between Truvada and Descovy and (ii) post-transition: sharing the experience of switching from Truvada to Descovy, or asking questions associated with the transition experience. In the context of PrEP drug transition, all discussions detected concerned a pathway of transition or switching from Truvada (pre-transition) to Descovy (post-transition).

**Figure 2:**
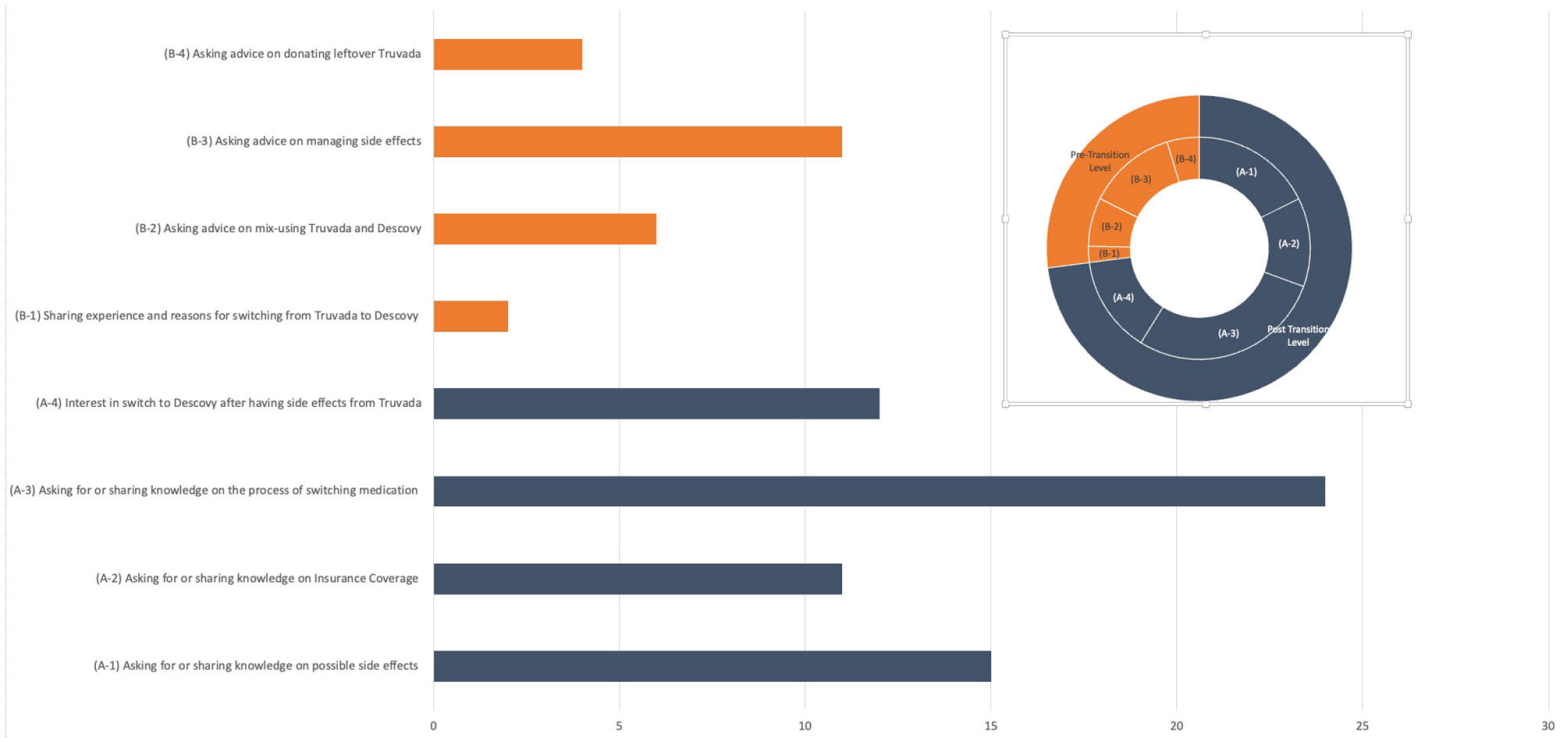
Content breakdown into sub-topics.

Among all signal posts, 62 posts (72.94% of total signal posts) were classified in the pre-transition parent topic (please see Figure 2 for the visual depiction of themes). We observed four main themes among these posts: conversations sharing or seeking knowledge on how to switch from Truvada to Descovy (A-3) that had the highest number and percentage of posts of all topics (*n* = 24, 22.86%); followed by topic (A-1) associated with users sharing or seeking knowledge on potential side effects from Descovy and also discussing reasons why this influenced their decision of not to engage in drug transition (*n* = 15, 17.65%); topic (A-4) included posts in which users expressed interest in switching to Descovy because of the purported side effects experienced from Truvada (*n* = 12, 28.23%); and topic (A-2) focused on sharing or seeking insurance coverage information associated with drug switching to a new PrEP treatment and how this impacted their decision to transition or not to transition to another PrEP treatment (*n* = 11,12.94%).

Among posts classified in post-transition parent topics (*n* = 23, 27.06%), we also observed four main themes. A major theme (B-3) that emerged was the discussion of side effect management after switching to Descovy (*n* = 11, 12.94%). Another topic (B-2) included discussions regarding mixing Truvada and Descovy medications (i.e., primarily focused on questions by users regarding how they should use (co-use) remaining Truvada medication after switching to and initiating Descovy) (*n* = 6, 7.07%). Topic (B-4) contained posts regarding users seeking information on how they can donate their remaining Truvada to other people who might benefit from prevention treatment access (*n* = 4, 4.71%). A final sub-topic (B-1) included general discussion of the experience of switching medications and why users switched to Descovy.

## 4. Discussion

Our study conducted a descriptive analysis of over 2,000 Reddit posts and found that just under 5% included specific discussions about transitions of use between Truvada and Descovy, with the vast majority of these posts originating from user-generated comments or posts related to SGM issues or from users self-identifying as members of the LGBTQ community, specifically gay and MSM populations. Key insights generated from these discussions include knowledge seeking and information sharing by users to help navigate decisions about transitioning to new PrEP therapy (pre-transition) and concerns that arose after switching from Truvada to Descovy (post-transition).

Our results add to insights generated by prior studies that have examined social media for clues regarding barriers and facilitators associated with PrEP, including specifically among gay and MSM populations. For example, a 2018 systematic review examined both peer-review studies and online posts to identify barriers and facilitators to PrEP among MSM, detecting six overarching categories that included online sources acting as facilitators to PrEP uptake and concerns about perceived side effects (20). A 2021 study specifically examined characteristics of PrEP-related content on Instagram for Truvada, including discussion by users about side effects (21). A recent 2022 study analyzed user perceptions and attitudes toward PrEP across multiple social media platforms (Twitter, YouTube, Instagram, Tumblr, and Reddit) and identified specific barriers to PrEP access including among minority online users (22). Finally, another 2022 study specifically examined conversations from Reddit users on PrEP from 2014 to 2019 and identified discussions about PrEP initiation and side effects as well as other themes (23).

Adding to these prior studies that examined social media for HIV and PrEP topics, this study was able to identify specific lived experiences regarding transitions of use for PrEP therapy, a topic subject to possible social stigma as it relates to HIV status, sexual orientation, and HIV-related risk behavior. However, due to Reddit's relative anonymity as an online platform and the ability to up and downvote content, it appears to be a platform where users may be more willing to discuss stigmatizing topics (26). Specifically, we observed that Reddit users actively shared their lived experiences with PrEP to aid in online peer-to-peer decision-making about the benefits and risks of PrEP therapy, including in the context of facilitating the transition of use or initiation of new HIV therapy. Importantly, users who may lack sufficient information to make informed decisions about PrEP therapy options may utilize these online communities as primary health information-seeking sources, even before or in the absence of consulting with a healthcare professional.

Furthermore, discussion of the impact of perceived side effects of different PrEP medications was prominent in the drug transition of use decisions, along with the discussion of access (e.g., insurance coverage) and cost. An additional consideration not specifically detected in this study but likely influencing the transition of use among users was the financial incentives associated with switching PrEP therapy. Descovy's regulatory approval for PrEP therapy occurred in 2019, just over 1 year prior to the generic availability of Truvada. At its introduction to the market, Descovy was offered with large commercial discounts which resulted in it launching at a 12% lower average net price than Truvada (27). These financial incentives led to changes in PrEP use. Specifically, within the first 9 months of it entering the market, nearly 30% of patients switched from Truvada to Descovy (28). In addition to the initial lower costs, patients may also prefer transitioning from Truvada to Descovy due to early reports that Descovy was less toxic and had fewer side effects, despite these claims since being refuted (10, 29). New themes that emerged from this study also included questions about the co-use of PrEP therapy and ways to donate unused medicines to other patients, unique in the context of examining behaviors associated with the transition of use for HIV/AIDS drugs.

Importantly, these findings provide insights that are important to post-market surveillance and pharmacovigilance efforts attempting to better understand perceptions of uptake, risk, and safety for PrEP among a community of predominantly gay and MSM users at higher risk for HIV. However, this study may not provide an extensive list of barriers and facilitators to accessing and adhering to PrEP therapy, as many people who contemplate HIV prevention or treatment may not openly discuss the unique complications they experience due to the stigmatization associated with this topic whether online or offline. Results can help inform how patients and specific gay and MSM populations in English-speaking countries experience and react to perceived adverse events and side effects and how it may impact the HIV care continuum of initiating, adhering to, and potentially transitioning between PrEP in preventing transmission (30). Importantly, the HIV burden remains high among African, Asian, and other non-English speaking countries. As such, most PrEP users were reported as residing in Africa in 2020 (31).

Hence, future studies should adopt methodologies used in this study for non-English language social media posts to better understand the unique barriers and facilitators in regions representing the largest HIV burden and greatest PrEP use, while also examining topics not explored in this study, such as the targeting of PrEP product advertising or outreach toward specific SGM populations. Future studies should also further validate results generated from social media with other sources of patient experiences (e.g., real-world data, prescribing and reimbursement information, and electronic health records) and integrate these novel data sources into a more proactive system of interdisciplinary pharmacovigilance to ensure better uptake and adherence to PrEP. In particular, the use of digital mixed methods that combine social listening approaches with in-depth qualitative data (e.g., focus groups) and robust quantitative data (e.g., surveys) designed specifically for at-risk SGM populations can better triangulate insights into PrEP barriers that can inform the development of future interventions and patient outreach.

## 5. Limitations

This study is primarily exploratory and descriptive in nature and has certain limitations. First, we only collected data from Reddit and limited our analysis to keywords and terms in the English language. Hence, the findings are not generalizable to all social media users who discuss transitioning between PrEP therapy, including those in other countries with higher HIV burdens but who are underrepresented in the population of Reddit users. Additionally, this study may represent an underestimate of people's experience with PrEP therapy and may not provide an extensive list of barriers to treatment due to the social stigma associated with HIV and discussing HIV-related care, including via online communities. Our collected and analyzed data sample size was also relatively small, which could lead to sampling bias. Hence, future studies should expand data collection and analysis approaches to different phrases and keywords associated with an individual's HIV-related risk behavior to obtain a more representative corpus of social media conversations and increase the sample size of data that can be analyzed. In addition, Reddit is a social media platform. There is a potential bias in using social media data including population biases, behavioral biases, content biases, and linking biases. Future studies should consider triangulating results from social listening approaches used in this study with other data sources (e.g., focus groups and survey instruments). Future studies should also expand data collection to additional platforms, languages, and specific keywords associated with HIV exposure and risk behavior to generate a more representative corpus of PrEP-related social media conversations.

## Data availability statement

The raw data supporting the conclusions of this article will be made available by the authors, without undue reservation.

## Ethics statement

All information collected during this study was available in the public domain, did not include any deleted posts, and the study did not involve any interaction with users. Any user indefinable information was removed from study results and any racial and/or ethnic identifiers were presented as aggregated results to ensure anonymity. This study has been approved by WCG Institutional Review Board's (WCGIRB's). WCG IRB is registered with the Office for Human Research Protections (OHRP) and FDA as IRB00000533.

## Author contributions

HG, QX, TJM, JL, and TKM jointly conceived the study, drafted the study, conducted data collection and analysis, and wrote and agreed to the final version of this manuscript.

## References

[1] *Global, HIV & AIDS statistics-−2020 fact sheet*. UNAIDS. Available online at: https://www.unaids.org/en/resources/fact-sheet (accessed March 5, 2021).

[2] Sullivan PS Giler RM Mouhanna F Pembleton ES Guest JL Jones J. Trends in the use of oral emtricitabine/tenofovir disoproxil fumarate for pre-exposure prophylaxis against HIV infection, United States, 2012–2017. Ann Epidemiol. (2018) 28:833–40. 10.1016/j.annepidem.2018.06.009 30037634 PMC6286244

[3] Hoen E't Berger J Calmy A Moon S. Driving a decade of change: HIV/AIDS, patents and access to medicines for all. J Int Aids Soc. (2011) 14:15. 10.1186/1758-2652-14-15 21439089 PMC3078828

[4] *Pre-Exposure Prophylaxis (PrEP)*. HIV Risk and Prevention. HIV/AIDS. CDC. Available online at: https://www.cdc.gov/hiv/risk/prep/index.html (accessed May 10, 2023).

[5] Schoenberg P Edwards OW Merrill L Martinez CA Stephenson R Sullivan PS. Willingness to use and preferences for long-acting injectable PrEP among sexual and gender minority populations in the southern United States, 2021–2022: cross-sectional study. J Int Aids Soc. (2023) 26:e26077. 10.1002/jia2.26077 36951057 PMC10034617

[6] de Aguiar Pereira CC Torres TS Luz PM Hoagland B Farias A Brito JDU. Preferences for pre-exposure prophylaxis (PrEP) among sexual and gender minorities: a discrete choice experiment in Brazil. Lancet Reg Health Am. (2023) 19:100432. 10.1016/j.lana.2023.100432 36950036 PMC10025414

[7] Grant RM Lama JR Anderson PL McMahan V Liu AY Vargas L. Preexposure chemoprophylaxis for HIV prevention in men who have sex with men. New Engl J Med. (2010) 363:2587–99. 10.1056/NEJMoa1011205 21091279 PMC3079639

[8] *Truvada Medication Information Sheet for Patients*. Available online at: https://www.cdc.gov/hiv/pdf/PrEP_GL_Patient_Factsheet_Truvada_English.pdf (accessed May 24, 2022).

[9] Blackwell CW Castillo HL. Human immunodeficiency virus pre-exposure prophylaxis: use of emtricitabine/tenofovir alafenamide. J Nurse Pract. (2021) 17:673–6. 10.1016/j.nurpra.2021.01.00233134420

[10] Mayer KH Molina J-M Thompson MA Anderson PL Mounzer KC Wet JJD. Emtricitabine and tenofovir alafenamide vs emtricitabine and tenofovir disoproxil fumarate for HIV pre-exposure prophylaxis (DISCOVER): primary results from a randomised, double-blind, multicentre, active-controlled, phase 3, non-inferiority trial. Lancet. (2020) 396:239–54. 10.1016/S0140-6736(20)31065-5 32711800 PMC9665936

[11] D'Angelo AB Westmoreland DA Carneiro PB Johnson J Grov C. Why are patients switching from tenofovir disoproxil fumarate/emtricitabine (truvada) to tenofovir alafenamide/emtricitabine (descovy) for pre-exposure prophylaxis? AIDS Patient Care STDS. (2021) 35:327–34. 10.1089/apc.2021.0033 34375141 PMC8380788

[12] Hill A Hughes SL Gotham D Pozniak AL. Tenofovir alafenamide versus tenofovir disoproxil fumarate: is there a true difference in efficacy and safety? J Virus Erad. (2018) 4:72–9. 10.1016/S2055-6640(20)30248-X 29682298 PMC5892670

[13] Kay ES Pinto RM. Is insurance a barrier to HIV preexposure prophylaxis? clarifying the issue. Am J Public Health. (2019) 110:e1–4. 10.2105/AJPH.2019.305389 31725314 PMC6893325

[14] Kimball D Rivera D Gonzales M Blashill AJ. Medical mistrust and the PrEP cascade among latino sexual minority men. AIDS Behav. (2020) 24:3456–61. 10.1007/s10461-020-02916-z 32405726 PMC7665998

[15] D'Avanzo PA Bass SB Brajuha J Gutierrez-Mock L Ventriglia N Wellington C. Medical mistrust and PrEP perceptions among transgender women: a cluster analysis. Behav Med. (2019) 45:143–52. 10.1080/08964289.2019.1585325 31343968 PMC6943929

[16] Wiginton JM Eaton LA Watson RJ Maksut JL Earnshaw VA Berman M. Sex-Positivity, medical mistrust, and PrEP conspiracy beliefs among HIV-negative cisgender black sexual minority men in Atlanta, Georgia. Arch Sex Behav. (2022) 51:2571–81. 10.1007/s10508-021-02174-7 34761347 PMC9085967

[17] Jaiswal J Halkitis PN. Towards a more inclusive and dynamic understanding of medical mistrust informed by science. Behav Med. (2019) 45:79–85. 10.1080/08964289.2019.1619511 31343962 PMC7808310

[18] Teng F Sha Y Fletcher LM Welsch M Burns P Tang W. Barriers to uptake of PrEP across the continuum among transgender women: a global scoping review. Int J STD AIDS. (2023) 34:299–314. 10.1177/09564624231152781 36793197

[19] Tekeste M Hull S Dovidio JF Safon CB Blackstock O Taggart T. Differences in medical mistrust between black and white women: implications for patient–provider communication about PrEP. AIDS Behav. (2019) 23:1737–48. 10.1007/s10461-018-2283-2 30264207 PMC7690288

[20] Hannaford A Lipshie-Williams M Starrels JL Arnsten JH Rizzuto J Cohen P. The use of online posts to identify barriers to and facilitators of HIV pre-exposure prophylaxis (PrEP) among men who have sex with men: a comparison to a systematic review of the peer-reviewed literature. AIDS Behav. (2018) 22:1080–95. 10.1007/s10461-017-2011-3 29285638 PMC5991474

[21] Walsh-Buhi E Houghton RF Lange C Hockensmith R Ferrand J Martinez L. Pre-exposure prophylaxis (PrEP) information on instagram: content analysis. JMIR Public Health Surveill. (2021) 7:e23876. 10.2196/23876 34061759 PMC8367150

[22] Xu Q Nali MC McMann T Godinez H Li J He Y. Unsupervised machine learning to detect and characterize barriers to pre-exposure prophylaxis therapy: a multiplatform social media study. JMIR Infodemiology. (2022) 2:e35446. 10.2196/35446 37113799 PMC10014091

[23] LoosierPSRenfroKCarryMWilliamsSPHogbenMAralS. Reddit on PrEP: posts about pre-exposure prophylaxis for HIV from Reddit users, 2014–2019. AIDS Behav. (2022) 26:1084–94. 10.1007/s10461-021-03463-x34536176

[24] Thomas DR. A general inductive approach for analyzing qualitative evaluation data. Am J Eval. (2006) 27:237–46. 10.1177/1098214005283748

[25] Terminology. DASH CDC. Available online at: https://www.cdc.gov/healthyyouth/terminology/sexual-and-gender-identity-terms.htm (accessed May 10, 2023).

[26] Amaya A Bach R Keusch F Kreuter F. New data sources in social science research: things to know before working with Reddit data. Soc Sci Comput Rev. (2021) 39:943–60. 10.1177/0894439319893305

[27] Dickson S Gabriel N Hernandez I. Estimated changes in price discounts for tenofovir-inclusive HIV treatments following introduction of tenofovir alafenamide. AIDS. (2022) 36:2225–7. 10.1097/QAD.0000000000003401 36205353 PMC9698192

[28] Hoover KW Zhu W Wiener J Huang Y-LA. Trends in Truvada and Descovy Prescriptions for PrEP in the United States, 2014–2020. Available online at: https://natap.org/2021/CROI/croi_201.htm (accessed December 15, 2022).

[29] Gupta SK Post FA Arribas JR Eron JJ Wohl DA Clarke AE. Renal safety of tenofovir alafenamide vs. tenofovir disoproxil fumarate: a pooled analysis of 26 clinical trials. AIDS. (2019) 33:1455–65. 10.1097/QAD.0000000000002223 30932951 PMC6635043

[30] HIV. Available online at: https://www.who.int/data/gho/data/themes/hiv-aids (accessed December 15, 2022).

[31] Global State of PrEP. Available online at: https://www.who.int/groups/global-prep-network/global-state-of-prep (accessed December 15, 2022).

